# “*We don’t normally require that in other contexts, do we*”: Interpersonal meanings of tag questions in British university seminars based on the BASE corpus

**DOI:** 10.3389/fpsyg.2022.1070937

**Published:** 2023-01-10

**Authors:** Lifang Wei, Alex Ho-Cheong Leung, Yufeng Sun

**Affiliations:** ^1^School of Foreign Languages, Shaoxing University, Shaoxing, China; ^2^Department of Humanities, Northumbria University, Newcastle upon Tyne, United Kingdom; ^3^College of International Education, Zhejiang University of Water Resource and Electric Power, Hangzhou, China

**Keywords:** tag questions, interpersonal meanings, British university seminars, BASE corpus, classroom interactions

## Abstract

This study investigates the interpersonal meanings expressed by English tag questions in the context of British university seminars from two dimensions: evidential modification of tags and conduciveness of responses. The data for the study derive from seminars in the British Academic Spoken English (BASE) Corpus, which is herein both quantitative and qualitative analyzed. The findings reveal that: (1) three-quarters of tag questions in the data are utilized by teachers, and the unmarked form of tag questions in university seminars are generally positive-negative forms, with a few examples of other varieties; (2) regarding evidential modification of tags, depending on the degree of epistemic certainty of the speakers on the proposition of the anchor, the default function of teachers’ tag questions in the data is to convey emphasis, followed by confirmatory function, with only a few cases of informational function; and (3) regarding conduciveness of responses, over 70% of tag questions are followed by no verbal response, while less than 30% are followed by explicit responses. Accordingly, these findings raise the question of whether tag questions can really provide sufficient scope for interactions in classroom. It is hoped that this nuanced, corpus-based analysis of tag question utilization within the context of British university seminars would empirically reveal the interpersonal relations between teachers and students and thereby shed light on more efficient seminar discussions.

## 1. Introduction

Tag questions are a common occurrence in English. They are defined as “utterances with an interrogative tag.” Huddleston and Pullum (2002, p. 891) identified the two defining components of tag questions as being an “anchor” and “tag.” For instance:

**Example 1:** *They are linked, aren’t they?*

In this example, “they are linked” is an anchor, whereas “aren’t they” is a tag, while the sentence as a whole represents a tag question.

This present study focuses on this canonical form of tag questions^1^ within the specific context of university seminars. The rationale underpinning the scope of this study is twofold. First, among prior relevant studies (to be discussed in detail in section “2 Literature review”), tag questions in the context of classroom interactions (including seminars) have yet to be systemically investigated, despite their frequent occurrence and the significance of their functions in classroom interactions. In particular, the way and extent to which tag questions contribute to interpersonal meanings, that is, “the ways that language choices establish a particular social relationship between the speakers and hearers” (Halliday, 1978, p. 112), remain under-researched. Second, by accessing the carefully recorded corpus data of the BASE Corpus, we have an opportunity to comprehensively dissect the idiosyncratic nuances of interpersonal meanings and how these contribute to classroom interactions. Within the broader context of the increasing internationalization of education, a systematic study of interpersonal meanings of tag questions drawing from the authentic seminar corpus of the BASE Corpus would provide an illuminating and helpful reference point for all university students, both British and international, in facilitating their awareness of the routine use of tag questions in seminar interactions, thus helping them to increase their linguistic repertoire *via* classroom participation. In addition, teachers also benefit from this study, insofar that the in-depth exploration of tag questions reveals the interactional structures and building of rapport in classroom seminars, which in turn sheds much-needed light on the nature of interactivity of tag questions. Seeking to develop our understanding in these areas, this present research analyzes the interpersonal meanings revealed through detailed corpus-based discourse analysis of both teachers’ and students’ use of tag questions in university seminars.

The following section reviews relevant academic literature related to tag questions and identifies the gaps in existing studies. In the third section, we detail the methods used for this study, explaining the data, and procedures used for analysis. Then, in the fourth section, we present the findings of this study, followed by a critical discussion of the findings. The final section concludes the research as a whole, before proposing potential avenues for further study.

## 2. Literature review

### 2.1. Previous studies on tag questions

Academic investigations focusing on tag questions in English have undergone several stages, including (but not limited to) a grammar-based approach, a pragmatic approach, a sociolinguistic approach, a prosodic analytical approach, an emphasis on diachronic study, and more.

The grammar-based studies of tag questions have tended to focus on ascertaining the illocutionary force of various types of tag questions based on formal variables, such as intonation and polarity. Such studies primarily position tag questions under the sub-types of “questions” which thereby separate anchor and tag. Quirk et al. (1985, p. 810), for instance, view tag question as “a further type of yes/no question which conveys positive or negative orientation.” This essentially focuses on tag questions with reversed polarity (i.e., the positive–negative type and negative–positive type), but tag questions with constant polarity (i.e., the positive–positive type and negative–negative type) are excluded. In addition, although studies of tag questions in this approach recognize that such reversed polarity tags betray the speakers’ bias toward the positivity or negativity of the proposition in the anchor, the findings nonetheless require significant refinement. For instance, more insights could be generated by analysis of the use of various forms of tag questions by incorporating real-life data, which would provide substantiation for the claims obtained from linguistic introspection.

Taking a different emphasis, pragmatic studies of tag questions predominately focus on the *functions* of tag questions. Pragmatic studies recognize that tag questions do not always function as purely response-seeking questions. For instance, Tottie and Hoffmann (2006) point out that “question use (informational types)” accounts for less than 3% of their data on tag questions. Other influential categories of tag question functions include Holmes (1995) and Algeo (1990), *inter alia*. Holmes (1995) classifies tag questions into two macro-categories—epistemic modal and affective—with the former referring to the modal functions while the latter consisting of facilitative, softening, and challenging functions. Before this, Algeo (1990) classified tag question functions as informational, conformational, punctuational, preemptive, and aggressive. Among these different labels of functions, four main function groups can be summarized: (1) to seek information; (2) to express politeness; (3) to intentionally use aggravating language (such as in a courtroom); and (4) to contribute to turn-taking. This is useful because it lays a foundation for the various types of functions of tag questions; however, these studies are not without their limitations. For instance, there are no clear definitions for all the labels of functions; there may be a convergence of interactional and stance meanings within and among these categories, thereby undermining the categorizations; moreover, due to the multi-functional nature of tag questions in specific contexts, it sometimes becomes difficult—if not impossible—to assign one specific function. More recently, Gómez González and Dehé (2020) have considered the pragmatics and prosody of tag questions, forging a new framework by which to map the form-and-function correlations of English tag questions based on four kinds of stance meanings (namely: epistemic, deontic, attitudinal, and textual). In so doing, their work provides new insights into this field of study.

The sociolinguistic approach to tag question studies encompasses gender studies as well as dialect and a variety of studies. Lakoff’s (1972) study reveals that women are primary users of tag questions, a finding that the author contends is reflective of their insecurity or lack of commitment. However, other studies generated different results. Dubois and Crouch (1975) state that men did use tag questions in both formal and informal contexts. Moreover, Aijmer (1996) investigates the use of tag questions by London teenage speakers, as well as other dialects across a variety of contexts, such as in Edinburgh-based speech, British and American English, Canadian English, and Asian English (including Hong Kong English).

A handful of studies also touch upon the prosodic analysis of tag questions, offering detailed surveys of the prosodic features of tag questions as part of a broader investigation into the functions associated with them, as well as the diachronic studies of tag questions (e.g., Tottie and Hoffmann, 2006).

### 2.2. Previous studies of classroom interactions and tag questions in classroom interactions

The past few decades have witnessed a resurgence of studies into classroom interactions, including Sinclair and Coulthard’s (1975) Initiation, Response, and Feedback structure, studies on language classrooms (Seedhouse, 2004; Fu, 2008; Yang, 2010; Lin and Leung, under review), functional approaches to classroom discourse (Christie, 2002), and critical analysis of classroom interactions (Kumaravadivelu, 1999). In particular, the analysis of question-and-answer communication formats has gained increasing attention, having been studied from a variety of dimensions, such as interactional structures, conversation analysis and corpus linguistics, and systemic functional approach (Sinclair and Coulthard, 1975; Long and Sato, 1983; Walsh, 2013; Yang, 2021; Yang and Yin, 2022).

Tag questions, as a prominent question form, play a significant role in classroom interactions, with seminars—as generally smaller, more conversational settings—being a particularly relevant context. Concomitant with the increase of international students in British tertiary education, international students encounter significant challenges in British university seminars. For instance, they are immediately at a disadvantage, being comparatively unfamiliar with normative British academic discourse, such as the process of participation, methods, and daily routines in university seminars, an unfamiliarity that arouses anxiety and impedes effective participation. Basturkmen (2016) points out that international students face challenges such as a lack of linguistic resources within classroom participation, with the use of tag questions being one such resource. Accordingly, it becomes necessary to study tag questions in the context of university seminars by clarifying the syntactic forms, as well as the pragmatic and interpersonal functions of tag questions, to promote the understanding of this form and identify ways of facilitating its flexible use in seminar participation.

In summary, previous studies on tag questions are relatively comprehensive, covering many contexts. Nonetheless, the study of tag questions in the context of university seminars remains hitherto understudied, despite its integral role in classroom interactions in terms of both knowledge transmission and the establishment of favorable interpersonal relations between participants. However, whether or not they create favorable conditions for opening interactions and discussions remains to be seen. Tag questions in university seminars are thus the object of focus in this present study.

### 2.3. Analytical framework

Interpersonal meaning, as proposed by Halliday (1978, p. 112), refers to “the participatory function of language” and is associated with how “language [is] organized as a resource for enacting roles and relations between the speaker and addressee” (Matthiessen et al., 2010, p. 126).

There is a broad academic consensus that tags convey interpersonal meanings (McGregor, 1995, 1997; Kimps, 2007, 2018; Axelsson, 2011; Kimps et al., 2014), and two layers of both subjective meaning and intersubjective meaning are denoted (McGregor, 1997). Subjective meaning primarily involves the speaker and proposition itself, whereas intersubjective meaning focuses on the speaker, hearer, and proposition. By using tag questions, the speaker wants to express a subjective meaning toward the proposition; on the contrary, the speaker simultaneously elicits an expected response to the proposition from the hearer. The first dimension has been referred to as the “evidential modification” of tags (McGregor, 1997, p. 222–233), or more expansively, that “tags modify the way in which the anchor relates to presuppositions and expectations.” Putting another way, tags are associated with the degree of certainty/epistemic commitment of the speaker and hearer vis-à-vis the proposition. The second dimension was referred to as the “conduciveness” of tags, primarily referring to tag questions indicating “which interactional position the speaker assumes in the dialogue and which response s/he expects from the hearer” (McGregor, 1997, p. 245).

A two-dimensional analytical framework extrapolating the interpersonal meanings of tag questions (based on McGregor, 1997; Kimps, 2018) is presented in Table 1.

**TABLE 1:** Two dimensions of interpersonal meanings of tag questions (based on McGregor, 1997; Kimps, 2018).

Dimensions	Key features	Description
Dimension I: subjective meaning	Evidential modification of tag questions (degree of speaker’s commitment to the proposition in the anchor).	Tag questions can modify the way in which the anchor relates to propositions and expectations (McGregor, 1997; Kimps, 2018).
Dimension II: intersubjective meaning	Conduciveness of tag questions (responses to tag questions).	Tag questions can indicate which interactional position the speaker assumes in the dialogue and which response s/he expects from the hearer (McGregor, 1997; Kimps, 2018).

Thus far, we have reviewed existing studies on tag questions and the nature of questions in classroom discourse, before introducing the analytical framework. In the following section, the methods of the study are introduced and explained.

## 3. Methods of the study

This small corpus-based study primarily adopts a qualitative discourse analysis approach (supplemented with concise quantitative data analysis to provide an overview of the findings). In this section, we will first present the research questions, followed by a detailed description of the data used for generating the findings of the study. Then, building upon the analytical framework for the study, the procedures for data coding and analysis will be explained thereafter.

### 3.1. Research questions

The overarching research question for this study is formulated as follows: In what ways are interpersonal relations revealed by teachers’ and students’ tag questions in British undergraduate university classroom interactions?

To answer this question, two sub-questions are investigated:

1) In what ways are subjective meaning presented in evidential modification by anchors of tag questions in British university undergraduate seminars?
2) In what ways are intersubjective meaning presented in the conduciveness of tags in British university undergraduate seminars?

### 3.2. Data description

The data analyzed in this study are derived from nine seminar sessions of the British Academic Spoken English (BASE) Corpus.^2^ The BASE project took place at the University of Warwick and the University of Reading between 2000 and 2005 (inclusive) under the leadership of Professors Hilary Nesi and Paul Thompson. The BASE Corpus consists of 160 lectures and 40 seminars recorded across a variety of departments, covering four broad disciplinary groups, each represented by 40 lectures and 10 seminars.

The BASE corpus was chosen for the current study for the following reasons. A large number of lectures and seminars are included in the corpus, and the quality of the data is also ensured, as the corpus was compiled by experienced professionals with funding from BALEAP, EURALEX, the British Academy, and the Arts and Humanities Research Council. There are no other alternatives freely and publicly available corpora for British seminars data at the time of research. The facilities and resources used to compile this publicly funded corpus were better than those that individual researchers could have obtained access to. The transcripts are available online and most of the video recordings are accessible upon request from the compilers. Compiling a large corpus of spoken data is a complicated process; efforts have to be put into ensuring the representativeness and size of the data, obtaining permission and access to suitable data, accessing professional equipment for video or audio recordings, transcription of the data, and so on. Therefore, utilizing the BASE Corpus, the most appropriate corpus available at the time of the research which has been used in other doctoral research and published journals, can save considerable time and energy. Considering all the above-mentioned factors, the BASE corpus was chosen to be used as English classroom data for the present study.

One of the potential drawbacks of using the BASE Corpus is that the data were recorded about two decades ago, which may not represent the latest trend in higher education. However, the basic two forms of instruction in classroom teaching included in the BASE corpus, lectures and seminars, are similar to the modes of teaching nowadays. In addition, seen from the long-term history of education, the time span for the data in the BASE Corpus can be regarded as a representation of the case for the early twenty-first century, thus still generating some insights into the current field of study.

Lectures and seminars are two forms of instruction settings in classroom teaching. On the whole, lectures are teacher-fronted monolog sessions, though sometimes with occasional questions or a few short discussions between the teacher and students. That is to say, the lectures of the BASE corpus are more formal and attach more importance to “transmission over negotiation” and “monolog rather than dialogue” (Hyland, 2009, p. 97–98) and involve “mainly a one-way form of communication.” In contrast, the seminars in the BASE corpus take various forms of interaction between the teachers and students, including whole-class discussion, group discussion, and students’ presentations, which are characterized by “explicit interactivity” (Hyland, 2009, p. 106) involving two-way communication, with participation from most of the people who are present, and they are less structured and more interactive.

Compared to the more monologic nature of the lectures, it seems that the seminar sessions provide a better platform for interaction with more examples of tag questions from the teachers and students. Therefore, the current data are drawn from nine sessions of seminars from three disciplines of the BASE Corpus (humanities, social science, and engineering), totaling 9.5 h and approximately 85,000 tokens. The details of the nine sessions of seminars chosen^3^ are presented in Table 2.

**TABLE 2:** Description of the data.

Code	Title	Duration (in minutes)	Words	Source
Session 1	Criminal law: Accomplice liability (law)	53′58″	8,755	sssem006
Session 2	Contemporary health issues: Unemployment and health (social policy and social work)	47′17″	7,495	sssem008
Session 3	The Cuban revolution (comparative American studies)	62′51″	11,198	ahsem003
Session 4	Beauty and “the thin red line” (film and television studies)	55′21″	8,950	ahsem006
Session 5	Radiation and photochemistry (chemistry)	55′35″	8,907	pssem001
Session 6	Questions and answers (engineering)	56′56″	9,037	pssem005
Session 7	“Built-in” social behaviors in territoriality and sexual behaviors (psychology)	37′26″	7,090	sssem007
Session 8	Introduction to health service (statistics)	43′58″	9,040	pssem008
Session 9	Curriculum English: Teaching short stories at key stage 2 (education)	97′38″	13,323	sssem004
Total		565′39″	83,777	

### 3.3. Data coding and procedures for analysis

**FIGURE 1:**
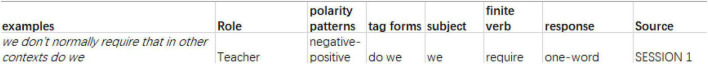
A screenshot of the coding of tag question examples in the Excel spreadsheet.

The first author first printed all the transcripts of the nine sessions and also used *AntConc*, a free and handy corpus analysis toolkit (Anthony, 2004), to extract all examples of variant tag questions from the data. Concordancer Tool in AntConc helped extract the examples of tag questions in the context while printing the data out facilitates manually checking the data at a granular level. Very few ineligible tag questions are removed, mainly including slips of tongues of speakers or incomplete tag forms. Then, all examples of extracted tag questions were put in an Excel spreadsheet, with relevant information such as roles (teacher or student), polarity patterns, tag forms, subject, finite verb, response (with response or no response), and source of the data recorded systematically. A sample screenshot of the spreadsheet is shown in Figure 1.

Following this, the first author analyzed the interpersonal meanings of tag questions from two dimensions: the first dimension related to the evidential modification of tags to the anchors by analyzing functions of tag questions in the current data (thereby building upon previous studies); and the second dimension derives from the conduciveness by analyzing responses to tag questions in the data.

To ensure the reliability of the study, during the coding and analysis of the data, the third author independently checked the coding, arriving at an agreement rate of 86%, and a few disagreements were solved through subsequent discussion.

## 4. Results and discussion

### 4.1. Overview of tag questions in the data

A total of 122 instances of tag questions are identified in the data sample. Among these, there are 99 examples of teacher-derived tag questions (accounting for 81.15%), while there are 23 examples of students’ tag questions (accounting for 18.85%). The distributions of teachers’ and students’ tag questions are shown in Table 3.

**TABLE 3:** Overview of teachers’ and students’ tag questions in the sample data.

Teacher tag questions	99 (81.15%)
Student tag questions	23 (18.85%)
Total	122 (100%)

**FIGURE 2:**
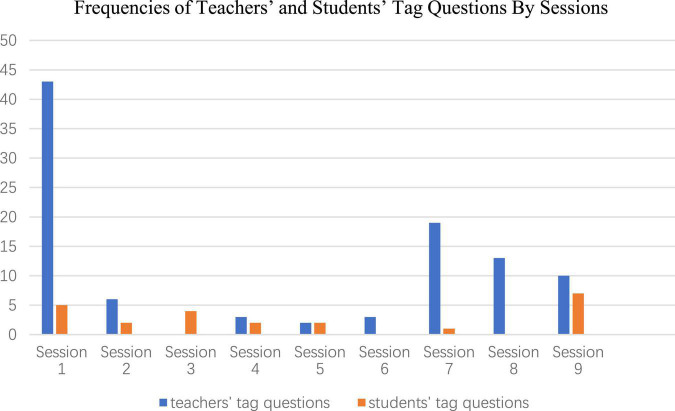
Frequencies of teachers’ and students’ tag questions across the sampled sessions.

Moreover, there are significant variances between different sessions, as illustrated in Figure 2, where it can be seen that in most of the sessions, teacher-derived tag questions occur more frequently than student-derived tag questions; and that Session 1 is the session containing most teachers’ tag questions, whereas Session 9 is the session where tag questions are mostly derived from the students. In Session 3, the teacher does not use tag questions at all, whereas in Session 6 and Session 8, students do not use any tag questions.

In terms of polarity, the data sample reveals that the vast majority of tag questions are reversed polarity tag questions (approximately 95%). Among them, positive–negative tag questions account for 80% (98 examples), with negative–positive tag questions accounting for approximately 14%, while positive–positive tag questions only account for a minor proportion (around 5%). Interestingly, there are no examples of negative–negative tag questions found in the data. In terms of roles of tag questions, teachers predominantly use positive–negative tag questions, with some instances of negative–positive questions, and a few positive–positive tag questions. On the other hand, students are found to mostly use positive–negative tag questions, with only two examples of negative–positive tag questions. No students have used positive–positive tag questions.

The distribution of forms of the polarity of teachers’ and students’ use of tag questions in the data is listed in Table 4.

**TABLE 4:** Polarity in teachers’ and students’ tag questions.

	Positive-negative	Negative-positive	Positive-positive
Teachers tag questions	77 (63.11%)	15 (12.30%)	7 (5.74%)
Students tag questions	21 (17.21%)	2 (1.64%)	0 (0%)
Total	98 (80.32%)	17 (13.93%)	7 (5.74%)

Such a distribution of tag question polarity is concordant with the findings of Kimps (2018), who examined tag questions in conversation, and identified a ratio of positive–negative tag questions (70%), negative–positive questions (18%), positive–positive tag questions (12%), and negative–negative tag question (0%). However, the data sample analyzed in this present study only utilizes declarative tag questions, whereas Kimps’ data sample comprising daily conversations (which is not based on classroom data) exhibited greater variety, with 14 instances of imperative tag questions and five instances of interrogative tag questions.

Following this preliminary overview of tag questions’ frequencies and ratios in the data, we shall now analyze the interpersonal meanings of tag questions from the two dimensions of evidential modification and conduciveness, as outlined in Section “2.3 Analytical framework”.

### 4.2. Interpersonal meanings of tag questions (1): Evidential modification of tag questions

In terms of evidential modification of tag questions, our findings reveal a continuum of degrees of certainty of epistemic commitment of the speaker toward the propositions in the anchor. These can be segmented into strong certainty, medium certainty, and low certainty. They carry different illocutionary forces, namely “please note,” “please check”, and “please tell.” Three categories can be created based on these: rhetorical tag questions, confirmational tag questions, and informational tag questions (examples to be provided in the following sub-sections). These relations are shown in Table 5.

**TABLE 5:** Interpersonal meanings of tag questions (1): Evidential modification.

Interpersonal meanings of tag questions (1): Evidential modification	Degrees of certainty	Illocutionary forces	Categories
	Strong	“Please noting”	Rhetorical tag questions
	Medium	“Please checking”	Confirmatory tag questions
	Low	“Please telling”	Informational tag questions

Table 6 shows that approximately three-quarters (73.77%) of the sample data consist of rhetorical tag questions. Among teachers-derived tag questions, it is found that teachers most frequently use tag questions for rhetorical effort (around 60%), whereas students also use rhetorical questions to assert their positions (13.93%); confirmational tag questions account for one-fourth (25.41%) of the sample data; and there is only one example for information-seeking purposes for which the teacher utilizes tag questions (0.82%).

**TABLE 6:** Strategies of teachers’ and students’ tag questions.

	Rhetorical	Confirmatory	Informational
Teacher tag questions	73 (59.84%)	25 (20.49%)	1 (0.82%)
Student tag questions	17 (13.93%)	6 (4.92%)	0 (0%)
Total	90 (73.77%)	31 (25.41%)	1 (0.82%)

Having presented a general picture of their distributions, we now conduct a more detailed examination of these categories, drawing from examples in the data.

#### 4.2.1. Rhetorical tag questions: Please noting

For rhetorical tag questions, the speaker exhibits significant certainty toward the proposition in his/her assertions. The function of this category is primarily to emphasize what is being said by the speaker, with the illocutionary force of “please noting.” Such category of tag questions has also been labeled as “punctuation tags” (Cheng and Warren, 2001, p. 1,431), which “function to emphasize or underline what is being said by the speaker” (ibid).

Our data sample reveals that both teachers and students use such rhetorical tag questions, with such tag questions tending to appear in the middle of the clauses, thereby not requiring any verbal response from the hearers.

All examples of this category in the data sample are tag questions with reversed polarity. Both negative–positive and positive–negative forms are identified, revealing a continuum of stronger degrees of certainty in terms of the speaker’s commitment toward the proposition in the anchor.

In the following two examples, the teachers use negative–positive forms of rhetorical tag questions to students:

**Example 8: (Session 1)**

**Table:** 

T:	… *it’s possible that could be a source of duty depends in*
	*a way (em) **there’s never been a case quite like this has***
	***there*** *but (eh) it’s quite possible*

This example is from Session 1, a seminar held by the Law Department discussing a legal case. Here, the teacher is quite certain of the statement in the anchor “there’s never been a case quite like this,” so he/she uses a tag question to emphasize this epistemic certainty, functioning as “please noting” that “there are no such cases like this,” a function which does not expect any response from the students, but facilitates the repetition or reinforcement of his/her former talk “it’s quite possible.”

The teacher does choose not to use a declarative clause here as a means of expressing the proposition most assertively. By adding tags at the end of the declarative clause, the teacher may be aiming to mitigate the assertive force of being too absolute in the proposition, thereby reducing the imposition of forcing students to accept his viewpoint. However, in practice, we can see that the teacher is certain of his knowledge, whereby the rhetorical use of the tag question skillfully delivers this knowledge to the students in a more assertive way. It demonstrates the teacher’s competencies in possessing knowledge while students take the role of receiving knowledge without any questioning. Therefore, this example reveals a tension between the “degree of certainty” of the informational content of the statement and the non-face-threatening motive, whereby a possible “conflict” of illocutionary and perlocutionary usage can be seen.

The following is another similar example:

**Example 9: (Session 1)**

**Table:** 

T:	… *the problem is that that **duty isn’t specified in the***
	***statutes is it*** *the statutes do create all sorts of duties (eh)*
	*relating to (eh) t- to most cars but not that one*

This example is also from Session 1, a law seminar. By employing this tag question, the teacher delivers the “emphatic meaning, underlying the preceding proposition.” Its usage reveals that the speaker (teacher) is the source of authority, and can direct how the interaction will move forward. However, in so doing, negotiation is closed down.

On the other hand, it emerges from the sample that students also use rhetorical tag requests when emphasizing their argument or evoking shared background knowledge with the teacher or with other students. In the following example, one student uses the positive–negative form of rhetorical tag questions to the teacher:

**Example 10: (Session 1)**

**Table:** 

**1**	*T:*	*Okay, why should he do enough to ensure the*
		*burglary takes place?*
**2**	*S4:*	*I don’t know [laughter] (hm) cos he’s I suppose*
		***he’s got the start of the details hasn’t he***
		*fo- for the crime and to ensure that he isn’t (em)*
		*liable as an accomplice*…

Example 10 (above) is from Session 1, a seminar in the Law Department discussing a specific legal case. In response to the teacher’s “why-question,” the student initially verbally acknowledges not knowing the answer. This might not be because of a lack of knowledge, but rather that she is simply uncertain and hence this acknowledgment can be employed as hedging the statement so that she is putting forward her thinking in a less face-threatening manner. After this prelude, the student uses a tag question “he’s got the start of the details hasn’t he” as the evidential grounding, with certain assertions, together with modality expression of “I suppose” to indicate her uncertainty and present her answer as potentially relevant in response to the teacher’s previous interrogative request. In this way, even if the student’s answer may not be correct, the face-threatening risk is mitigated.

#### 4.2.2. Confirmatory tag questions: “Please checking”

Confirmatory tags function “to invite the addressee to agree with the speaker or to draw the addressee into discourse by providing support to the addressee” (Cheng and Warren, 2001). This usage is explained by Mithun (2012, p. 2071) as “an appeal for acknowledgment of shared knowledge, experience, or values, as speakers seek to establish a bond with their listeners.”

Our data sample reveals examples where teachers employ positive–negative tag questions, negative–positive tag questions, and positive–positive tag questions to confirm with students. In these examples, a degree of strong-to-medium certainty can be found in terms of the speaker’s commitment toward the proposition in the anchor, depending on the specific context of the interaction.

The following is an example of a positive–negative tag question found in the sample data:

**Example 11: (Session 7)**

**Table:** 

1	*S8:*	*like with the chickens how they have bigger things*
		*on their heads (eh)*
2	*T:*	***cones they’re called cones I think aren’t they***
3	*S8:*	*yeah*

This example is from Session 7, a seminar hosted by the Psychology Department. In this positive–negative tag question, the teacher expresses little doubt *via* the proposition in the anchor; and by using the tag question “they’re called cones I think aren’t they,” the teacher attempts to provide support to the student while exhibiting a strong certainty toward the proposition in the anchor. Meanwhile, the use of the modality expression “I think” is likely used to serve as an appeal for involvement in this shared knowledge with students.

The following is an example of negative–positive tag questions used to confirm with students:

**Example 12: (Session 1)**

**Table:** 

1	*T:*	… *(em) Ducross is liable because he should*
		*have interfered with her*
2	*S4:*	*(hm)*
3	*T:*	*prevented her from committing the crime and*
		***we don’t normally require that in other***
		***contexts do we***
4	*S4:*	*No*

This example derives from Session 1, a seminar in the Law Department. The teacher employs this tag question with negative polarity to gain confirmation from the students on the statement in the anchor “we don’t normally require that in other contexts.” It is also noteworthy that the teacher here uses the inclusive plural “we” as the subject, demonstrating a clear intention to establish a measure of solidarity between him and the students. The negative polarity in the anchor “don’t” conveys the bias of an expected negative answer toward this question. Finally, this tag question is followed by the response from a student, “no,” indicating his/her agreement.

Example 12 thus reveals that the teacher exerts the power to initiate the bias of an expected response by utilizing different forms of polarity within the tag question. In this specific example, the teacher conveys a combination of simultaneous solidarity building and influence assertion. On the surface, the teacher uses such tag questions to project “an inclusive approach to decision making,” but actually, it reinforces the power of the teacher in that their real intention is to invite the students to accept the teacher’s argument. Accordingly, by probing into the seeming solidarity of the relationship between the teacher and the students, an actual asymmetrical relationship can be identified between them.

A positive–positive tag question is also employed by the teacher to confirm responses with students, as is shown in the example below:

**Example 13: (Session 7)**

**Table:** 

1	S8:	*and humans have a choice I suppose animals*…
		*hasn’t got the choice (xx) humans tend to*
		*choose their partners*
2	T:	***you feel you feel humans have a choice do you***
3	S8:	*Yeah*
4	T:	*right yeah that’s a good point humans have a*
		*choice you can go your own way*…

This example comes from Session 7, a seminar from the Psychology Department, in which students are discussing the differences between animals and humans. S8 puts forward her viewpoint relating to the latter’s ability to choose their partners, after which, the teacher utilizes a constant polarity tag question, “you feel humans have a choice do you.” Differing from the previous example, this use of tag question does not convey the opinions of the speaker, but rather, it is used as a reported statement to echo the previous student’s viewpoint. This usage corroborates Nässlin (1984), who claims that the use of constant polarity tag questions indicates no personal opinion of the truth of the proposition of the anchor, whereas the use of reverse polarity tag questions implies that the speaker believes that the proposition in the anchor is true.

In this example, the teacher, by choosing to employ this constant positive tag question, maybe making an attempt to obtain confirmation from the student on her contribution of ideas.

On the other hand, students in our data also make use of tag questions for confirmatory purposes, both to confirm an expected action and for checking information on certain knowledge points.

**Example 14: (Session 3)**

**Table:** 

1	*S1:*	*so in fact name x and name x go and swap*
2	*S5:*	***I should swap shouldn’t I***
3	*S3:*	*yeah it might be an idea*

Example 14 is from Session 3, a debate seminar on the success or failure of the Cuban Revolution. It occurs at the beginning of the class when the teacher and the students are discussing whether and how to rearrange their seats for the convenience of their discussion. This example thus focuses on discussing the action of “swapping seats.” S1 suggests S5 and S3 go and swap, after which S5 uses a tag question “I should swap shouldn’t I” to request confirmation from S1, which is affirmed by S3, “yeah.” S5 then swapped his seat.

In this example, the subject is “I” and the finite is “should,” which are repeated in the tag. The first-person subject reveals that this example is not a request to ask other people to do some action, but is rather related to the action of “swapping” to be realized by himself, as a response to the previous suggestion of S1, “name x (S5) and name x (S3) go and swap.”

This example reveals the negotiation between students over seat swapping to facilitate the convenience of a group debate, and in this process of interaction, a subtle relationship between the students is established. S1 first proposes that S5 and S3 swap seats, whereby S5 successfully utilizes a tag question with the modality “should” (as the finite) to check his obligation to swap seats. Interestingly, this tag question is not responded to by S1, from whom the proposal derives, but by S3, who responds with the positive answer “yeah” and the modulated declarative “it might be an idea,” in which the low-value modality of “might” mitigates the force carried in the former proposal by S1. If S5 were responded to by S1 (i.e., the person who made the proposal), it may seem that S1 exerts a measure of control over his classmate. In avoiding this potentiality, a more equal relationship is built between the students *via* a certain degree of mitigation.

Another example used by students to confirm certain knowledge points is presented below:

**Example 15: (Session 5)**

**Table:** 

1	*T:*	*no okay well right an- any offers on w- on the*
		*methyl chloride one*
2	*S17:*	***I think you just add radiation that would split***
		***from this wouldn’t it***
3	*T:*	*sorry the*
4	*S17:*	*just add the radiation would split (xx) a radical*
5	*T:*	*yeah a- if you can have solid methyl chloride you*
		*probably would get (em) some (em) disruption*
		*like*

This example is from Session 5, a seminar held by the Chemistry Department. S17 employs a tag question “*I think you just add radiation that would split from this* ***wouldn’t it***” as a tentative response to the teacher’s question. In essence, the student possesses some measure of certainty in her proposition, demonstrated by her use of the anchor here, though she remains not completely sure. Accordingly, some strategies (including modality expressions “I think” and “would”) are included, to perform hedging functions through which the student can show her uncertainty. Similarly, the use of the tag “wouldn’t it” also reinforces her doubt and undermines her certainty of the statement. The tag also functions as a way to elicit confirmation from the teacher, who is supposed to have authority in curriculum-related knowledge. Additionally, it also serves as a potential face-saving strategy if the answer is wrong.

#### 4.2.3. Information-seeking tag questions: “Please telling”

Tag questions for real information-seeking purposes are primarily used to obtain information from the addressee and are classified as an informational type of function (Algeo, 1990; Tottie and Hoffmann, 2006), more specifically, as an “epistemic modal function” by Holmes (1995). In essence, they express genuine uncertainty of the speaker, which is to say, they are genuine questions with no assumption of knowing the expected response the hearer would or could provide. When speakers employ tag questions to elicit responses, they do so by being uncertain about the information in the anchor and seeking a response from the addressees, thereby exhibiting low certainty toward the proposition in the anchor. Such uses are not frequent in our data, which corroborates the quantitative findings of Tottie and Hoffmann’s (2006) study, in which the informational type of tag questions only accounts for a minority proportion (less than 3%).

**Example 16: (Session 1)**

**Table:** 

1	*T:*	… ***the next seminar is after Easter isn’t it***
2	*S1:*	*is it we don’t have a seminar this week*
3	*T:*	*i don’t think we do*

Example 16 is from Session 1, a seminar from the Law Department. It occurs at the end of the class when the teacher uses a tag question to request information from the students, specifically the “time for the next seminar.” The teacher may have some idea of the date of the seminar, but he is not certain, so he uses this tag question to elicit information from the students, which is responded to by a student who questions “is it we don’t have a seminar this week.” Accordingly, as for the information sought in the tag question, “time for the next seminar,” the teacher believes that the addressees (the students) may have better, or at least, similar, knowledge on this point, justifying his use of this tag question.

This example reveals that when negotiating the topic of “the time for next seminar,” which is not subject-related knowledge, both the teacher and the students are supposed to have similar knowledge. The use of the tag question by the teacher may represent an attempt to institute a more equal power relationship between the students and himself.

#### 4.2.4. Summary

In summary, based on the criteria of evidential modification of tags to propositions in the anchor, our research sample reveals that tag questions can be classified into three types: strong certainty, medium certainty, and low certainty, which in aggregate form a continuum from rhetorical tag questions for emphasis (“please noting”), confirmatory tag questions to invite the addressee to confirm the statement (“please checking”), to genuine information-seeking tag questions (“please telling”). This continuum also reflects the different levels of knowledge status held by different speakers.

Our analysis reveals that most teacher-derived tag questions in English university seminars are used as rhetorical requests to emphasize a certain point of knowledge. Such use of tag questions functions as part of broader assertion strategies for “coercing agreement” (Cheng and Warren, 2001) rather than indicating tentativeness. This finding helps explain why most of the rhetorical tag questions are teacher-derived, thereby originating from the more powerful participant in the classroom interaction.

In addition, this research also finds that most of the rhetorical uses of tag questions among teachers concern curriculum-based knowledge, of which the teachers enjoy greater epistemic power compared to the students. However, when it comes to other issues not closely related to curriculum-based knowledge (such as the time for the next seminar, see Example 16), teachers employ tag questions to seek information, helping to establish a more equal relationship between them and the students. As indicated in the examples in this section, different kinds of power-neutralizing and solidarity-building relationships between the teacher and students can be perceived in a specific communicative context with regard to different goals of interaction. The flexible use of multiple strategies involving tag questions reveals the multi-institutional role of the teacher as a controller, guide, facilitator, primary knower, and so on.

Moreover, students in English university classrooms are also found to utilize tag questions to fulfill diverse functions, for instance, asserting their argument by citing some facts as evidence. This reveals that students can express their epistemic knowledge more confidently by flexible utilization of tag questions.

After exploring the first dimensions of interpersonal meanings of tag questions from the perspective of evidential modification, we now move to the second dimension: the conduciveness of tag questions.

### 4.3. Interpersonal meanings of tag questions (2): Conduciveness of tag questions

In using tag questions, a speaker not only expresses a degree of commitment toward the proposition in the anchor as discussed but also indicates an attitude toward the hearer. More specifically, the conveyance of tag questions reveals how the speakers hope to affect turn allocation, as well as how the speakers perceive the exchanges and expected contribution of the hearers. This constitutes the second dimension of interpersonal meanings of tag questions, which will now be discussed.

In our data sample, the speakers’ utilization of particular forms of tags expresses a conduciveness toward the expected response of the hearers, and it is also a powerful tool by which to control turn allocation. Martin and White (2005, p. 102) contend that tag questions may be seen as indicating “potential negotiation,” and this negotiation may either be “expanded” or “contracted.” More specifically, when the negotiation is expanded (i.e., opening up), tag questions become response-eliciting; yet when the negotiation is contracted (i.e., closed down), no response is sought, and such tag questions are referred to as rhetorical.

In terms of the conduciveness of tag questions, the findings herein demonstrate the following patterns. Three types of response are found in the data: no response, one-word response, and discretionary response. Correspondingly, these create no space, little space, or more space for negotiation by either holding the floor or giving the floor to the addressee. This is summarized in Table 7.

**TABLE 7:** Interpersonal meanings of tag questions (2): Conduciveness of tag questions.

Interpersonal meanings of tag questions (2): Conduciveness of tag questions	Degrees of space for negotiation	Control of turn allocation	Types of response
	No space	Floor-holding	No response
	Little space		With response (one-word)
	More space	Floor-giving	Discretionary response

Table 8 provides a general overview of the responses to tag questions, as revealed in the sample data.

**TABLE 8:** Responses to tag questions in the data.

	No response	With response
Teacher tag questions	73 (59.84%)	26 (21.31%)
Student tag questions	16 (13.11%)	7 (5.74%)
Total	89 (72.95%)	31 (27.05%)

Approximately three-quarters (72.95%) of tag questions receive no response; only a quarter (27.05%) is followed by a response.

Of those with responses, the majority are expected confirmatory responses (the majority of which are one-word responses), with only a very small number of discretionary responses. This reveals a general trend of preference for alignment, and that there are not many opportunities for the negotiation of meanings. Analysis of detailed examples is presented in the following two sub-sections.

#### 4.3.1. No verbal response

Most of the rhetorical tag questions are found to be the first type of expected non-verbal response. For example:

**Example 17: (Session 1)**

**Table:** 

*T:*	… *I think in relation to his death you said well it’s*
	*probably manslaughter **it doesn’t have to be does it***
	*could be murder*…

This example is from Session 1, a law seminar. The negative polarity in the anchor of the tag question here “it doesn’t have to be (manslaughter)” implies a negative bias toward the expected negative answer. This substantiates Kimps (2007, p. 272) who states that “[i]t is … generally accepted that tag questions convey the speakers’ orientation to the proposition by signaling a specific attitude and the expected response.” In this example, as feedback to the student’s previous answer, the teacher does not utter the answer directly, but utilizes a tag question with negative polarity to hint at the expected negative answer; meanwhile, this approach softens the force, making it more acceptable to (or less imposing for) the students. The expected response as an alternative to the student’s previous answer may have already formed in the minds of the students, whereby the subsequent utterance of the teacher “could be murder” reinforces this expected answer. The use of modality expressions such as “probably” and “could be” further encourage students to think from multiple perspectives.

In this way, even with no explicit verbal responses being expected, the teacher may still successfully evoke answers in the minds of the students. In this case, usually little or no time is left for the hearer to take turns; consequently, no space for negotiation is created, inhibiting further discussion.

#### 4.3.2. With response

For this type of response to tag questions, two sub-types are identified in our data sample: one-word response and discretionary response. Relevant examples will now be discussed.

##### 4.3.2.1. One-word response from the hearer

Our data sample yields many one-word responses such as “yes” or “no,” used to confirm or deny, which is concordant with Newbury and Johnson (2006, p. 221) who have found that tag questions are “specifically intended to prompt a respondent to confirm or deny a version of events presented in the question.” In other words, responses to tag questions are either to confirm or deny. Moreover, Cameron et al. (1988, p. 87) maintain that “if a question contains a completed proposition, this takes more interactive work to challenge than it does to consent to; the consequence is that respondents tend to produce confirmations of the embedded proposition”. This implies that the preferred responses to tag questions are confirmational, and it is a phenomenon confirmed in our present data sample.

One-word responses to tag questions (to show confirmation) manifest in varying forms, depending on the polarity of tags. For instance, for the positive–negative tag questions, the response to show confirmation is “yeah” (see Example 11); while for the negative–positive tag questions, the response to show confirmation is “no” (see Example 12):

**Example 11: (Session 7)**

**Table:** 

1	S8:	*like with the chickens how they have bigger*
		*things on their heads (eh)*
2	T:	*cones **they’re called cones I think aren’t they***
3	S8:	*Yeah*

**Example 12: (Session 1)**

**Table:** 

1	*T:*	… *(em) Ducross is liable because he should*
		*have interfered with her*
2	*S4:*	*(hm)*
3	*T:*	*prevented her from committing the crime and*
		***we don’t normally require that in other***
		***contexts do we***
4	*S4:*	*No*

In Example 12 (Session 1), a seminar from the Law Department, the negative polarity in the anchor of the tag here (“we don’t…”) implies the negative answer for this tag question. The one-word response is obtained from one student, “no.” Superficially, the use of tag questions “gives the addressee leeway, not forcing him to go along with the view of the speaker” (Lakoff, 1972, p. 54) by utilizing a request, rather than a statement. However, upon closer inspection, it is confirmed that “the very construction of a tag question suggests that the speaker has certain assumptions and is biased toward a certain answer” (Tsui, 1992, p. 92). As such, the speaker *expects* (and is seen to expect) a negative answer to this tag question.

There is a possible cross-linguistic difference in terms of responses to negative–positive tag questions, such as when delivered in English and Chinese. More specifically, in the English example, the response “no” mainly echoes the negative polarity “don’t” in the proposition in the anchor, while the response “yes” in the Chinese example mainly reinforces the idea of the agreement, “yes, I agree with you.” Therefore, special attention needs to be paid to the responses to negative tag questions in English and Chinese.

It is also notable that in this type of one-word response, despite there being a change of floor taking place, the space for negotiation remains relatively small, and no further space for dialogue or extended discussion is created.

##### 4.3.2.2. Discretionary response

Some of the responses to tag questions are not the expected responses implied in the polarity of tags (as seen in Example 16, explored above). Example 18 is another pertinent example.

**Example 18: (Session 1)**

**Table:** 

1	*T:*	*okay what would you say about bill*
2	*S8:*	*well (em) he was aware that the burglary*
		*to be taking place so in that sense (xxx) crime*
		*was going taking place so he’s the first one*
3	*S7:*	***he did phone the police didn’t he*** *to [warn them*
4	*S8:*	*[it says that albert did is that a mistake*
5	*S7:*	*that’s a mistake*
6	*T:*	*yeah that’s a mistake sorry a mistake of mine*…

Example 18 is from Session 1, a law seminar. The student, S7, employs a tag question “he did phone the police didn’t he” to emphasize his assertion of the fact in the legal case, using the marked form of the finite verb “did” in the anchor. However, this is interrupted by an overlapping speech from S8, “it says that Albert did…,” after which S8 then uses another polar request to ask “is that a mistake.” Therefore, in this example, as a challenge to the assertion of S7 in the form of a tag question, S8 cites the information in the handout as evidence, using this to pose a contrary opinion, though later confirmed as a mistake in the handout by S7 and the teacher.

Thornbury and Slade (2006, p. 119) contend that “[e]xpected responses support the proposition of the speaker and thereby serve to create alignments and solidarity. By contrast, the discretionary responses are either disengaging and non-committal or openly confronting.” Accordingly, discretionary responses to tag questions create more space for negotiation and extended discussions.

#### 4.3.3. Summary

In summary, the responses to the tag questions in English university classroom settings range from expected non-verbal responses, positive or negative verbal responses indicated in the polarity of the anchor part of the tag questions, to discretionary responses in relation to specific classroom interaction contexts.

Generally speaking, most of the tag questions identified herein are followed by no verbal response or a simple one-word confirmatory response, indicating that not much space for negotiation is created. Discretionary responses, which can open up more space for negotiation, are found in limited quantities in our data sample. Therefore, responses to tag questions in the data are predominantly floor-holding, rather than floor-giving, and tend to show more alignment than disalignment.

### 4.4. Discussion: Interpersonal meaning and tag questions

The interpersonal meanings of tag questions in the seminar sessions have been collated and summarized in Table 9.

**TABLE 9:** Two dimensions of interpersonal meanings of tag questions.

Interpersonal meanings of tag questions				
Dimension I: evidential modification	Degree of certainty		Illocutionary force	Categories
	Low certainty	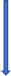	‘Please telling”	Informational tag questions
	Medium certainty		“Please checking”	Confirmatory tag questions
	Strong certainty		“Please noting”	Rhetorical tag questions
Dimension II: conduciveness	Degree of space for negotiation		Control of turn allocation	Types of response
			Floor-giving	With response
	More space	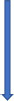		(Discretionary response)
	Little space			(One word response)
	No space		Floor-holding	No response expected

As demonstrated in Table 9, regarding the first dimension of evidential modification, the degrees of speakers’ certainty toward the propositions contained within the anchors form a continuum from low, medium, to strong certainty. The degrees along this continuum correspondingly realize the three categories of informational, confirmatory, and rhetorical questions, with the illocutionary force of “please telling,” “please checking,” and “please noting,” respectively. On the second dimension of conduciveness of tag questions, various types of responses emerge: discretionary response, one-word response, and no response. These also form a continuum in terms of space for negotiation, ranging from more space, little space, to no space, with the corresponding floor-giving to floor-holding attributes in terms of control of turn allocation.

Equally pertinent, the dual role of tag questions cannot be ignored. By using requests in the form of tag questions, teachers may be attempting to involve students in class discussions; on the other hand, the conducive forms of tag questions also indicate that teachers can utilize tag questions to influence, control, or otherwise guide students to think toward certain expected directions (i.e., the correct answers). This dual function of tag questions supports the findings of Harres (1998, pp. 113–114) in that “tag questions used for their facilitative function are a cooperative strategy aimed at reducing social distance and expressing solidarity or support. As part of their coercive or challenging function, tag questions force addressees to respond to and agree with the speaker (conducive tag questions).”

In this way, the use of tag questions plays an important role in the simultaneous realization of interactional involvement and communicative or pedagogical control in classroom interactions. More specifically, on one hand, tag questions function as an important tool to establish rapport with students and to encourage involvement, for it seemingly gives students a chance to participate in classroom interactions by presenting their comments or correcting any misconceptions. However, whether these opportunities are realized by students is a totally different matter.

This finding is very important to help illustrate the dynamics of interpersonal meanings enacted in the use of tag questions by teachers and students in the classroom (which is different from Brown and Levinson’s (1987) static and pre-established view of power and distance relations) in classroom interactions. The use of tag questions in university seminars reflects a dynamic interplay of both epistemic power and deontic power between teachers and students. Epistemic power is related to the right to know, describe, and access (Kent, 2012). The role of teachers might be expected to claim epistemic power for their experience or professional knowledge in certain subjects, but the roles of teachers cannot always guarantee epistemic power. It might be the case that certain students claim to have more knowledge in certain points, with the necessity to negotiate “the legitimacy of claims in these cases” (Barker and Annerstedt, 2014, p. 5). Deontic power is related to which party can set the rules concerning what should be done (Craven and Potter, 2010). Teachers can exercise their deontic power by allocating tasks to their students in accordance with their lesson plans. However, in a similar vein, their attempt to exercise deontic power cannot be expected to be observed by students all the time; instead, deontic power is an “interactional accomplishment, claimed, displayed and negotiated” (Stevanovic and Peräkylä, 2012, p. 315).

The current study has focused on some of the mechanisms of interpersonal relations in terms of evidential modification of tags and conduciveness based on the negotiation of roles and identities in classroom interactions, which are realized in specific linguistic expressions of tag questions in classroom discourse. It is important to note that the semiotics of interpersonal meanings is very complex and involve comprehensive and unexhaustive components.

## 5. Conclusion

### 5.1. Summary of the study

Analyzing data from the BASE Corpus, our research confirms that both teachers and students in British university seminar contexts utilize tag questions, whereby the former uses tag questions far more frequently than the latter. The unmarked form, that is, the most frequent form, used by both teachers and students is the positive–negative form, while teachers also exhibit greater use of the negative–positive as well as the positive–positive forms. Students meanwhile only occasionally employ the negative–positive form, with modality expressions often used together with tag questions.

In terms of the interpersonal meanings carried *via* tag questions at the dimension of evidential modification, depending on the degree of epistemic certainty of the speakers on the proposition of the anchor, functions of tag questions are found to form a continuum from rhetorical tag questions, confirmatory tag questions, to informational tag questions. In our data sample, rhetorical tag questions are found to account for almost three-quarters of tag questions in the data, demonstrating strong certainty of the speaker toward the proposition in the anchor, whereas confirmatory tag questions are found to account for around one-quarter, which reveals the moderate certainty of the speaker toward the proposition in the anchor, and insinuates an aim of checking with the hearer. Only one instance of a tag question is found to be a real information-seeking question. In this sense, we can conclude that based on the data analyzed herein, most tag questions are utilized to capture the attention of the hearer, rather than opening up a dialogue by seeking specific missing information from the hearer.

In terms of interpersonal meanings of tag questions at the dimension of conduciveness, three-quarters are identified as receiving no verbal response, while one-quarter are followed by a response, most of which comprise expected (one-word) confirmatory responses. This may indicate that even though certain types of tag questions have the potential to open up a negotiation between teachers and students, the opportunities for true interactions vary significantly. Whether and how tag questions are employed in class to stimulate interactions (perhaps in a communicative-oriented classroom) is thus worth further exploration.

In addition to the above two dimensions, the dual functions of tag questions are a conducive means for teachers to maintain control in the classroom activities or, while concurrently building rapport, align themselves with the students. This finding substantiates Harres (1998, pp. 113–114) who concludes that: “tag questions … are a cooperative strategy aimed at reducing social distance and expressing solidarity or support… [and also] coercive or challenging, [for they] force addressees to respond to and agree with the speaker.”

### 5.2. Implications of the study

This investigation into the interpersonal meaning of tag questions in English university seminars from the two dimensions of evidential modifications of tags and conduciveness has several implications, both theoretically and practically.

In terms of theory, our study demonstrates the potential for further refinement of the analytical framework of interpersonal meanings. More specifically, future studies are encouraged to explore question types (such as polar interrogatives and declarative questions) that are presently omitted by prevailing theoretical frameworks.

Practically, this study can offer referential support to teachers and students in the UK. By ascertaining and understanding detailed interpersonal use contexts, they can make more effective use of tag questions in their communications, persuasion strategies, engagement, and involvement strategies to manage turn allocation more effectively in seminar interactions, thereby facilitating learner gain. With the empirical support provided by corpus-based studies similar to ours, teachers can benefit from a greater awareness of the relationship between the use of tag questions and the actual effect of creating and closing dialogue space with students. This, in turn, enables teachers to better balance/control engagement and build meaningful rapport through classroom interactions. Moreover, it is hoped that international students who come to study in the UK can benefit from greater awareness of differences in academic cultures in terms of using and responding to tag questions. Being equipped with such an awareness can enable international students to better integrate themselves into the seminar interactions in the British academic community.

Furthermore, this study offers meaningful insights for English for Academic Purpose (EAP) course designers and the utilization of corpus materials in education. When designing course books for international students, it is beneficial for educators and designers to refer to actual examples in the corpus (such as the BASE Corpus) to better tailor suggestions for the international student body. When it comes to tag questions, drawing on real examples from the corpus can more effectively highlight varied uses of tag questions in terms of form, function, and potential responses, thereby facilitating a more comprehensive understanding of the interpersonal meanings underpinning tag questions. This is in line with the emphasis on the significance of using clear and tested examples of corpus-based data in English language teaching (Viana, 2022).

### 5.3. Limitations and directions for future study

Given the size of our dataset, future research is encouraged to verify the findings reported herein against more comprehensive data from related contexts, such as university seminars in other countries.

Future studies may also include more sessions of seminars from various disciplinary schools, thereby allowing for the identification and comparison of potentially disciplinary-specific characteristics. Other specific features, such as prosodic features of tag questions (which are also important for the interpretation of tag questions) and detailed conversational features (such as turn positions), are recommended for inclusion. Moreover, the influence of various factors on the interpersonal meanings of tag questions, such as types of activities in seminars (e.g., debating and problem-solving activities), requires further exploration. Finally, examining the use of tag questions in seminars through a cultural lens (rather than linguistics) as well as the interpersonal meanings revealed therein also offer a fruitful avenue for future research.

## Data availability statement

Publicly available datasets were analyzed in this study. This data can be found here: https://www.reading.ac.uk/acadepts/ll/base_corpus/.

## Author contributions

LW was in charge of data coding and analysis and writing the draft of the manuscript. AH-CL was in charge of commenting on the draft of the manuscript and helped clarify aspects of the analyses. YS helped check the coding of the data analysis and proofreading of the manuscript. All authors contributed to the article and approved the submitted version.

## Footnotes

1 Invariant tag questions like “*That’s the point, right*” are not included in the current study.

2 Special thanks go to Professor Hilary Nesi who kindly granted us access to the data of the BASE Corpus.

3 In total, 20 seminars for PG level students are excluded because our study focuses on undergraduate level university classrooms. Stage performances or project meetings between students with two teachers were also excluded from our study. In addition, special thanks go to Dr. Alex Nicholson and Professor Regina Weinert, who helped us double check the transcriptions of the English data in the BASE corpus.
